# Revisiting the relationship between regenerative ability and aging

**DOI:** 10.1186/1741-7007-11-2

**Published:** 2013-01-21

**Authors:** Ashley W Seifert, S Randal Voss

**Affiliations:** 1 Department of Biology, University of Kentucky, Lexington, KY 40506, USA; 2 Department of Biology, University of Florida, Gainesville, FL 32601, USA; 3 Spinal Cord and Brain Injury Research Center, University of Kentucky, Lexington, KY 40506, USA

## Abstract

Contrary to the longstanding view that newts (*Notophthalamus viridescens*), but not axolotls (*Ambystoma mexicanum*), can regenerate a lens, a recent report in *BMC Biology *by Panagiotis Tsonis and colleagues shows axolotls indeed possess this ability during early larval stages. In contrast, they show that zebrafish never posses this ability, even as embryos. This underscores the importance of comparing regenerative ability across species and reinforces the need to consider organ regeneration in the context of evolution, development, and aging.

See research article: http://www.biomedcentral.com/1741-7007/10/103

## Commentary

New insights in regenerative biology will continue to arise from studies of animal models that present a diversity of regenerative responses. To better leverage these models, it will be important to consider regenerative ability within the context of evolution, life history, physiology, and development [1]. Here we highlight a long-standing problem, the loss of regenerative ability as an organism ages. Considering this fundamental relationship between aging and regeneration in naturally regenerating systems may help translate new discoveries into effective applications in regenerative medicine.

While humans possess varied and diverse mechanisms for physiological regeneration - regeneration to maintain and renew organ functions throughout life - this renewal declines with age; furthermore, we possess a very limited ability for the regeneration of tissues following injury. Enviously, we watch flawless regeneration of limbs, lens, retina, spinal cord, brain, heart, and neuro-sensory cells, seemingly throughout life, by distant vertebrate relatives. There are, however, limits to the ability of any organism to regenerate, and understanding these limits may be the key to understanding why humans cannot regenerate whole organs. Although examples of tissue regeneration are rare among birds and mammals, tissue regeneration occurs in amphioxus, the most basal living chordate [2]. This suggests that humans likely share mechanisms that are used by salamanders and other vertebrates to regenerate organs. Salamanders provide a good stepping-off point for considering factors that limit regenerative ability because they present a basal tetrapod condition that most closely approximates ancestral lineages leading to amniotes.

## Regenerative potential is generally higher during early life stages

**Figure 1:**
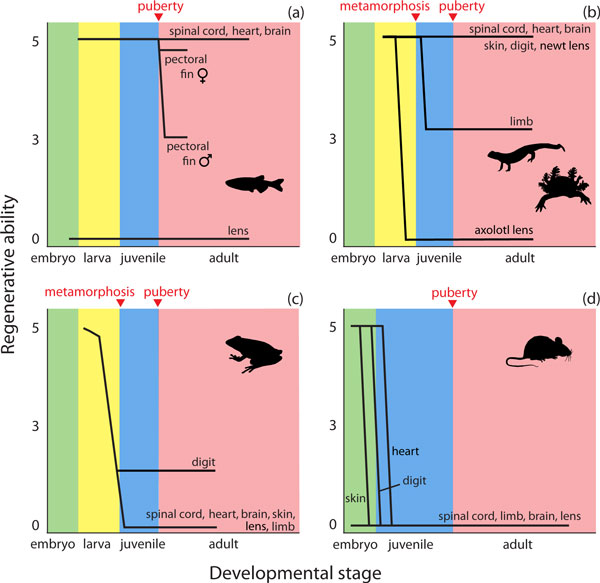
**Reparative regeneration as a function of developmental stage among vertebrate models of regeneration**. Panels depict how the regenerative ability of homologous structures varies across ontogeny for the primary vertebrate models of regeneration. Regenerative ability is presented on a 0 to 5 scale; five represents perfect regeneration and zero no regeneration. Major ontogenetic stages are represented as embryo, larva, juvenile and adult with metamorphosis, puberty or both indicated for each species. **(a) **Zebrafish exhibit lifelong regenerative capacity of spinal cord, brain, and heart. They cannot regenerate a lens at any time in development and while pectoral fins (homologous to tetrapod limbs) regenerate in juveniles, their regenerative capacity is reduced following puberty, with females retaining a higher capacity for complete regeneration. **(b) **Salamanders and newts are the archetypical tetrapod regenerator, retaining near perfect regeneration of most organs and appendages well into adulthood (although almost no studies have tested these abilities in old animals). They do, however, experience a decline in limb regeneration following metamorphosis, which usually manifests as patterning defects and loss of limb elements. Axolotls can only regenerate lenses as early stage larvae. **(c) **Although most frogs exhibit some degree of regeneration as larvae, with the exception of limited digit and very restricted limb regeneration in some species, they do not exhibit regenerative ability as adults. **(d) **Mammals exhibit some regenerative capacity as embryos but lose almost all of this ability before or shortly after birth for the structures listed.

To understand why and how regenerative ability varies across ontogeny and phylogeny requires a comparative approach that evaluates regeneration of homologous structures throughout development, preferably using species that diverged from a common ancestor. Unfortunately, few rigorous studies have examined regeneration throughout development. Still, ontogenetic changes in regenerative potential have been described for a few species and several lines of evidence support the idea that embryos, larvae, and juveniles of many species have greater potential for tissue repair and regeneration than adults (Figure 1). Frogs and mice, for example, are capable of regenerating skin during early life stages but lose this ability later in development, while axolotls retain the capacity for skin regeneration even after metamorphosis ([3] and references therein). Similarly, the ability of zebrafish to regenerate pectoral fins and frogs to regenerate limbs is reduced in older animals, while heart regeneration appears unaffected even in very old fish ([1,4] and references therein). Adding to these studies, a report by Suetsugu-Maki *et al*. [5] now shows that axolotls can regenerate a lens during early life stages, while most vertebrates cannot regenerate lenses at all. And yet, newts are capable of lens regeneration throughout life and if the progenitor cells that restore lenses in non-regenerating species, including humans, are cultured *in vitro*, cells with lens phenotypes differentiate [6]. Thus, while the potential for lens regeneration is apparently a shared derived trait of vertebrates, that potential is restricted during development in most species. Together, these examples suggest that even in some salamanders with boundless regenerative abilities, constraints imposed by aging, either at the physiological or cellular level, work to limit regenerative capacity in organ- and species-specific manners.

## What limits regenerative ability during aging?

Understanding how aging changes cells, tissues, and physiological systems is key to identifying mechanisms that limit regenerative ability. During both development and regeneration, relatively undifferentiated cells become specified to form organs that then undergo tremendous growth, but the overall process differs between the two. Regeneration is activated in response to injury, depends upon tissue-specific progenitor cells, and occurs under physiological conditions and within an extracellular environment that differs from the embryonic state. Embryonic cells have great potential for cellular reprogramming, and cellular reprogramming through epigenetic modifications and changes in transcription are associated with regenerative responses [7]. During development, cells exhibit changes in transcription that limit signaling pathways associated with cellular plasticity. For example, cells differentiate and lose the ability to enter the cell cycle, both of which must be reversed for limb regeneration to occur in salamanders. The maintenance of plastic cellular states and cell-cycle re-entry are likely associated with the actions of tumor suppressor proteins like retinoblastoma protein (RB), the levels and activation states of which are known to vary during regeneration in salamanders and across developmental stages in mammals [8,9]. Taken together, studies suggest that the abundances of key regulatory molecules are permissive for cell cycle re-entry from a quiescent state in young life stages, but restrictive as an organism ages, and this limits regenerative ability. Future studies that quantify levels of such molecules in young and old salamanders may shed new light on progenitor cell activation, regenerative ability, and potentially, diseases of aging such as cancer.

## How do systemic factors affect regenerative ability?

Early, heightened cellular plasticity in response to injury reflects not only local but also systemic factors that are difficult to disentangle without ontogenetic perspective. The importance of systemic factors is dramatically shown in studies of parabiotic mice that differ in age but share the same circulatory system. Serum from young animals stimulates older muscle to regenerate and serum from old individuals decreases the regenerative capacity of young muscle [10]. A similar phenomenon was noted in the ability of a young systemic milieu to rejuvenate aged oligodendrocyte precursor cells and promote remyelination of axons in old mice [11]. While these studies provide further support for the idea that regenerative potential correlates negatively with aging, they also show that regeneration is not simply a local property of cells and tissues. Instead, regeneration also depends upon blood cells and serum factors that have broad access to tissues and progenitor cell niches - and the properties of these cells and factors change during development.

Many animals undergo post-embryonic growth and developmental phases that commence in response to circulating hormones that are released at relatively specific times during ontogeny. In the case of amphibian metamorphosis, thyroid hormone (TH) reprograms juvenile cells and activates adult progenitor cells, and this brings about the conversion of tadpole aquatic larvae into more terrestrial adults. Interestingly, while newts always undergo metamorphosis, axolotls rarely do unless treated exogenously with TH [12]. Future studies that use hormones to induce metamorphosis at different times during ontogeny may be able to disentangle the effects of aging from intrinsic regenerative ability, as it has already been shown that prolonging the larval state enhances regenerative ability compared to same-aged animals that have undergone metamorphosis [13].

## Regeneration insight from comparative approaches

In coming years, we envision a new, golden age in regenerative biology. Animals present a diversity of regenerative responses that vary across organs, developmental stages, and phylogeny. Increasingly, advances in genetic and genomic technologies will make it possible to compare regenerative responses within and among animal models to identify factors that cause regenerative ability to change with aging.

## References

[B1] Seifert AW Monaghan JR Smith MD Pasch B Stier AC Michonneau F Maden M The influence of fundamental traits on mechanisms controlling appendage regeneration Biol Rev 2011 87 330 345 21929739 10.1111/j.1469-185X.2011.00199.x

[B2] Somorjai IM Somorjai RL Garcia-Fernandez J Escriva H Vertebrate-like regeneration in the invertebrate chordate amphioxus Proc Natl Acad Sci USA 2012 109 517 522 10.1073/pnas.1100045109 22203957 PMC3258630

[B3] Seifert AW Monaghan JR Voss SR Maden M Skin regeneration in adult axolotls: a blueprint for scar-free healing in vertebrates PLoS ONE 2012 7 e32875 10.1371/journal.pone.0032875 22485136 PMC3317654

[B4] Itou J Kawakami H Burgoyne T Kawakami Y Life-long preservation of the regenerative capacity in the fin and heart in zebrafish Biol Open 2012 1 739 746 10.1242/bio.20121057 23213467 PMC3507221

[B5] Suetsugu-Maki R Maki N Nakamura K Sumanas S Zhu J Del-Rio Tsonis K Tsonis PA Lens regeneration in axolotl: new evidence of developmental plasticity BMC Biol 2012 10 103 23244204 10.1186/1741-7007-10-103 PMC3554534

[B6] Eguchi G Eguchi G, Okada TS, Saxen L Cellular and molecular background of Wolffian lens regeneration Regulatory Mechanisms in Developmental Process 1988 Amsterdam: Elsevier 147 158 10.1016/0922-3371(88)90111-6 3061589

[B7] Yakushiji N Yokoyama H Tamura K Repatterning in amphibian limb regeneration: A model for study of genetic and epigenetic control of organ regeneration Semin Cell Dev Biol 2009 20 565 574 10.1016/j.semcdb.2008.12.007 19146968

[B8] Tanaka EM Gann AA Gates PB Brockes JP Newt myotubes reenter the cell cycle by phosphorylation of the retinoblastoma protein J Cell Biol 1997 136 155 165 10.1083/jcb.136.1.155 9008710 PMC2132456

[B9] Levi BP Morrison SJ Stem cells use distinct self-renewal programs at different ages Cold Spring Harb Symp Quant Biol 2008 73 539 553 10.1101/sqb.2008.73.049 19150957

[B10] Conboy IM Conboy MJ Wagers AJ Girma ER Weissman IL Rando TA Rejuvenation of aged progenitor cells by exposure to a young systemic environment Nature 2005 433 760 764 10.1038/nature03260 15716955

[B11] Ruckh JM Zhao JW Shadrach JL van Wijngaarden P Rao TN Wagers AJ Franklin RJ Rejuvenation of regeneration in the aging central nervous system Cell Stem Cell 2012 10 96 103 10.1016/j.stem.2011.11.019 22226359 PMC3714794

[B12] Johnson CK Voss SR Salamander paedomorphosis: linking thyroid hormone to salamander life history and life cycle evolution Curr Top Dev Biol 2013 release date 02/01/13 10.1016/B978-0-12-385979-2.00008-3 23347521

[B13] Gibbs KM Chittur SV Szaro BG Metamorphosis and the regenerative capacity of spinal cord axons in *Xenopus laevis* Eur J Neurosci 2011 33 9 25 10.1111/j.1460-9568.2010.07477.x 21059114

